# Supplementary material for: Endoprosthetic Reconstruction in Ewing’s Sarcoma Patients: A Systematic Review of Postoperative Complications and Functional Outcomes
Source: J Clin Med. 2022 Aug 8;11(15):4612. doi: 10.3390/jcm11154612 (PMC9370018; doi:10.3390/jcm11154612)
Supplement: Supplementary file 1 [file jcm-11-04612-s001.zip › jcm-1833726-supplementary.pdf]

Table S1. Summary of post-operative complications

Characteristic	Number
**Type I Failure: Soft Tissue**	
**Restricted ROM**	6
**Wound healing complication**	2
**Joint instability (including dislocations)**	5
**Nerve lesion**	1
**Superficial infection**	5
**Type II Failure: Aseptic loosening**	3
**Type III Failure: Structural**	
**Mechanical Failure**	2
**Periprosthetic Fractures**	1
**Type IV: Deep Infection**	9
**Type V: Local Tumour Progression**	3
**Other Complications**	
**Amputation**	4
**Total complications**	41

Table S2. Demographics of individual patients with Ewing's sarcoma across the 17 chosen studies.

Case	Study	Gender	Age	Location of Tumour	Prosthesis used	Other treatment	Complication
**1**	Benevenia 2015 [14]	**-**	16	Proximal Humerus	Repiphysis®	Neoadjuvant chemotherapy	Contracture
**2**	Benevenia 2015 [14]	**-**	11	Distal femur	Repiphysis®	Neoadjuvant chemotherapy	Contracture, Deep infection
**3**	Vijayan 2011 [15]	F	4	Distal femur	Stanmore	Neo adjuvant chemotherapy	Joint instability, flexion deformity
**4**	Torner 2016 [16]	F	12	Proximal femur	MUTARS®	Neo adjuvant + adjuvant chemotherapy	-
**5**	Hanna 2010 [17]	M	25	Femoral diaphysis	Stanmore	Neo adjuvant chemotherapy + radiotherapy	-
**6**	Hanna 2010 [17]	M	12	Femoral diaphysis	Stanmore	Adjuvant chemotherapy	-
**7**	Hanna 2010 [17]	F	10	Femoral diaphysis	Stanmore	Neo adjuvant chemotherapy	Deep infection
**8**	Yang 2017 [18]	F	26	Distal tibia	Stanmore	-	-
**9**	Schiller 1995 [19]	M	9	Distal tibia	HMRS	Neo adjuvant chemotherapy	Deep infection, joint instability
**10**	Dotan 2010 [20]	n/a	6	Proximal femur	Kotz	Neoadjuvant chemotherapy + adjuvant chemotherapy + radiotherapy	Superficial infection (2), deep infection, contracture, dislocation, mechanical failure, wound dehiscence
**11**	Dotan 2010 [20]	n/a	8	Proximal femur	Kotz	Neoadjuvant chemotherapy + adjuvant chemotherapy + radiotherapy	Dislocation, aseptic loosening
**12**	Dotan 2010 [20]	n/a	14	Proximal femur	Kotz	Neoadjuvant chemotherapy + adjuvant chemotherapy + radiotherapy	-

**13**	Dotan 2010 [20]	n/a	9	Proximal tibia	Kotz	Neoadjuvant chemotherapy + adjuvant chemotherapy + radiotherapy	Contracture
**14**	Dotan 2010 [20]	n/a	11	Distal femur	Kotz	Neoadjuvant chemotherapy + adjuvant chemotherapy + radiotherapy	Deep infection
**15**	Dotan 2010 [20]	n/a	11	Proximal femur	Kotz	Neoadjuvant chemotherapy + adjuvant chemotherapy + radiotherapy	Aseptic loosening, superficial infection, peri prosthetic fracture
**16**	Dotan 2010 [20]	n/a	11	Proximal femur	Kotz	Neoadjuvant chemotherapy + adjuvant chemotherapy + radiotherapy	Superficial infection, deep infection
**17**	Dotan 2010 [20]	n/a	14	Proximal femur	Kotz	Neoadjuvant chemotherapy + adjuvant chemotherapy + radiotherapy	-
**18**	Dotan 2010 [20]	N	9	Distal femur	Kotz	Neoadjuvant chemotherapy + adjuvant chemotherapy + radiotherapy	-
**19**	Dotan 2010 [20]	n/a	10	Proximal femur	Kotz	Neoadjuvant chemotherapy + adjuvant chemotherapy + radiotherapy	-
**20**	Dotan 2010 [20]	n/a	7	Proximal tibia	Kotz	Neoadjuvant chemotherapy + adjuvant chemotherapy + radiotherapy	Deep infection, contracture, mechanical failure, aseptic loosening

**21**	Dotan 2010 [20]	n/a	13	Proximal femur	Kotz	Neoadjuvant chemotherapy + adjuvant chemotherapy + radiotherapy	Deep Infection, amputation due to local recurrence
**22**	Yoshida 2011 [21]	M	12	Proximal tibia	Kotz	Neo adjuvant + adjuvant chemotherapy and radiotherapy	Amputation due to local recurrence
**23**	Shekkeris 2009 [22]	F	42	Distal tibia	Stanmore	Neo adjuvant + adjuvant chemotherapy and radiotherapy	-
**24**	Shekkeris 2009 [22]	M	15	Distal tibia	Stanmore	Neo adjuvant + adjuvant chemotherapy and radiotherapy	-
**25**	Raciborska 2021 [23]	M	6	Distal tibia	MUTARS	Neo adjuvant chemotherapy + adjuvant chemotherapy	-
**26**	Raciborska 2021 [23]	M	18	Distal tibia	MUTARS	Neo adjuvant chemotherapy + adjuvant chemotherapy	-
**27**	Raciborska 2021 [23]	M	15	Distal tibia	MUTARS	Neo adjuvant chemotherapy + adjuvant chemotherapy	-
**28**	Raciborska 2021 [23]	M	13	Distal tibia	MUTARS	Neo adjuvant chemotherapy + adjuvant chemotherapy	-
**29**	Yoshida 2008 [24]	F	8	Distal femur	Stanmore	Neo adjuvant chemotherapy	-
**30**	Yoshida 2008 [24]	M	7	Distal femur	Kotz	Neo adjuvant chemotherapy	-
**31**	Yoshida 2008 [24]	M	12	Proximal tibia	Kotz	Neo adjuvant chemotherapy	-

**32**	Erol 2021 [25]	F	38	Periacetabular	LUMiC	Neo adjuvant chemotherapy + adjuvant chemotherapy	-
**33**	Erol 2021 [25]	F	34	Periacetabular	LUMiC	Neo adjuvant chemotherapy + adjuvant chemotherapy	Amputation due to local recurrence
**34**	Erol 2021 [25]	M	34	Periacetabular	LUMiC	Neo adjuvant chemotherapy + adjuvant chemotherapy	Dislocation
**35**	Erol 2021 [25]	F	36	Periacetabular	LUMiC	Neo adjuvant chemotherapy + adjuvant chemotherapy	-
**36**	Erol 2021 [25]	F	46	Periacetabular	LUMiC	Neo adjuvant chemotherapy + adjuvant chemotherapy	-
**37**	Erol 2021 [25]	F	17	Periacetabular	LUMiC	Neo adjuvant chemotherapy + adjuvant chemotherapy	-
**38**	Puri 2012 [26]	M	17	Femoral diaphysis	RESTOR	Adjuvant chemotherapy	-
**39**	Ayoub 1999 [27]	F	10	Proximal humerus	Stanmore	Chemotherapy + Radiotherapy	-
**40**	Ayoub 1999 [27]	F	8	Proximal humerus	Stanmore	Chemotherapy + Radiotherapy	Amputation due to pain
**41**	Ayoub 1999 [27]	M	11	Proximal humerus	Stanmore	Chemotherapy + Radiotherapy	-
**42**	Ayoub 1999 [27]	M	7	Proximal humerus	Stanmore	Chemotherapy + Radiotherapy	Wound dehisence
**43**	Ayoub 1999 [27]	F	11	Proximal humerus	Stanmore	Chemotherapy + Radiotherapy	-
**44**	Ayoub 1999 [27]	M	8	Proximal humerus	Stanmore	Chemotherapy + Radiotherapy	-
**45**	Ayoub 1999 [27]	M	6	Proximal humerus	Stanmore	Chemotherapy + Radiotherapy	Radial nerve palsy

**46**	Ayoub 1999 [27]	F	10	Proximal humerus	Stanmore	Chemotherapy + Radiotherapy	-
**47**	Wafa 2015 [28]	F	15	Proximal humerus	Stanmore	Neo adjuvant + adjuvant chemotherapy	-
**48**	Wafa 2015 [28]	F	32	Proximal humerus	Stanmore	Neo adjuvant + adjuvant chemotherapy	-
**49**	Wafa 2015 [28]	F	9	Proximal humerus	Stanmore	Neo adjuvant + adjuvant chemotherapy	Superficial wound
**50**	Wafa 2015 [28]	F	8	Proximal humerus	Stanmore	Neo adjuvant + adjuvant chemotherapy	-
**51**	Wafa 2015 [28]	F	12	Proximal humerus	Stanmore	Neo adjuvant + adjuvant chemotherapy	Periprosthetic infection
**52**	Wafa 2015 [28]	F	7	Proximal humerus	Stanmore	Neo adjuvant + adjuvant chemotherapy	-
**53**	Wafa 2015 [28]	F	10	Proximal humerus	Stanmore	Neo adjuvant + adjuvant chemotherapy	-
**54**	Ji 2019 [29]	M	10	Distal femur	Non hinged CCK	Neo adjuvant + adjuvant chemotherapy	-
**55**	Ji 2019 [29]	M	9	Distal femur	Non hinged CCK	Neo adjuvant + adjuvant chemotherapy	-
**56**	Lewis 1986 [30]	F	10	Femoral Diaphysis	Lewis	Neo adjuvant + adjuvant chemotherapy and radiotherapy	-
**57**	Lewis 1986 [30]	F	13	Femoral diaphysis	Lewis	Neo adjuvant + adjuvant chemotherapy and radiotherapy	-
